# A case of cranium bifidum with meningocele in Ayrshire calf

**DOI:** 10.1186/s12917-016-0936-9

**Published:** 2017-01-13

**Authors:** Mosiany Letura Kisipan, Caleb Oburu Orenge, David Njogu Gacheru, Raphael Muchangi Ngure

**Affiliations:** 1 Department of Veterinary Anatomy and Physiology, Faculty of Veterinary Medicine and Surgery, Egerton University, P.O. Box 536–20115, Egerton, Kenya; 2 Department of Veterinary Clinical Studies, Faculty of Veterinary Medicine and Surgery, Egerton University, P.O. Box 536–20115, Egerton, Kenya

**Keywords:** Cranium bifidum, Meningocele, Skull vault, Occipital, Interparietal, Veterinary intervention, Developing world

## Abstract

**Background:**

Congenital cranial bone defects predispose to herniation of meninges, sometimes with brain tissue involvement, to form a cerebrospinal fluid (CSF)–filled cyst in the head. Such defects mainly results from focal failure of neural tube closure during fetal development and has been reported in various species of domestic mammals.

**Case presentation:**

A one week old Ayrshire calf with a fluctuant swelling on parieto-occipital region of the head was referred to the faculty. The calf was always lying on lateral recumbency and exhibited resistance to deep palpation around the swelling and neck flexion. Embedded to the midline of the dorso-caudal surface of the cyst’s wall was a hard longitudinally oriented structure. The case was diagnosed as meningocele by means of radiographic examination. As the likelihood to full recovery was greatly reduced due to the negative impact already meted on brain tissue by intracranial pressure, the calf was euthanized on grounds of animal welfare and the diagnosis confirmed by anatomopathological findings which also revealed a circular bone defect in parieto-occipital region of the skull vault and a flattened bony structure embedded to the cyst’s wall.

**Conclusion:**

Anatomopathological findings confirmed the diagnosis as cranial bifidum with meningocele at the parieto-occipital region of the skull vault. The presence of a bony structure embedded to the wall of meningeal sac was rather unusual and could not be sufficiently explained. It was however thought to, most likely, represent a part of interparietal bone that failed to get incorporated into squamous part of occipital bone as a result of the defect. The report also highlights challenges that work against timely delivery of urgent veterinary interventions in rural set ups of Africa and rest of the developing world, often leaving veterinarians with animal welfare consideration as main determinant of intervention measures.

## Background

The vault of mammalian skull is constructed of frontal, parietal and a contribution from squamous part of occipital bone. These bones develop from different ossification centers derived from tissues of different embryonic origins [1–5]. The squamous part of occipital bone develops from early fusion of interparietal and supraoccipital bones. The interparietal bone in turn develops from fusion of different element derived from distinct embryonic tissues, the mesoderm and neural crest cells [1, 6–8].

Neural tube defects (NTD) are congenital malformations of the central nervous system resulting from failure of the neural tube closure during embryogenesis [9]. Cranial bifidum is one of the NTD that manifests as a focal bone defect in the cranium usually with protrusion of meninges to form a CSF-filled sac-like swelling called meningocele. In some instances, the protrusion may also include brain tissue thus called encephalomeningocele [10–13]. The morphogenesis of these bone defects is not simply due to defective ossification but have instead been reported to depend on primary neural tube defect whereby, focal neural tube dehiscence from embryonic ectoderm fails leading to a focal failure in the development of skeletal encasement [13–15]. Either or both the genetic and environmental factors have been associated with the development of these congenital defects [11, 14–16].

Cranial meningocele has been described in humans and a number of domestic animal species, apparently being more common in cattle compared to other domestic animals [12, 16, 17]. The defects vary in size but are always related to suture line [15].

## Case presentation

A one week old male Ayrshire calf with a swelling on parieto-occipital region of the head was referred to the Faculty of Veterinary Medicine and Surgery of Egerton University. From history, the swelling was initially smaller at birth but progressively increased in size. The calf’s appetite was normal and was bottle-fed by the owner.

**Fig. 1:**
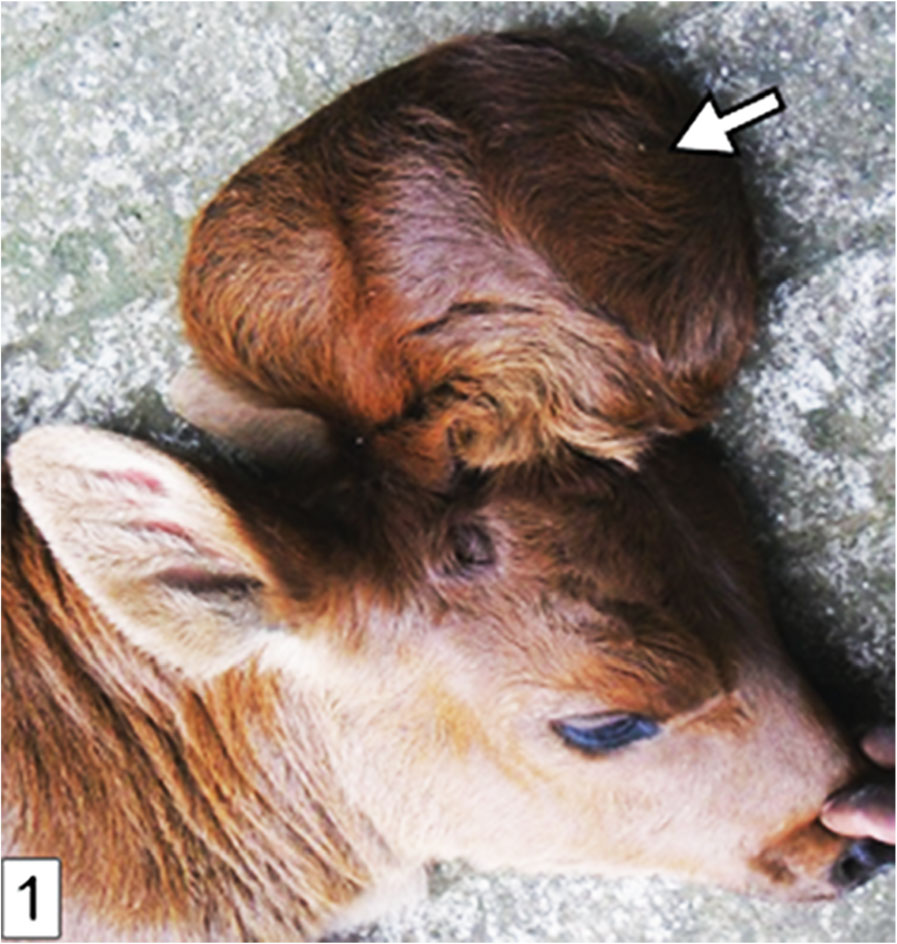
A photo of indisposed calf with protrusion (*open arrow*) covered by hairy skin on the occipital region of the cranium

**Fig. 2:**
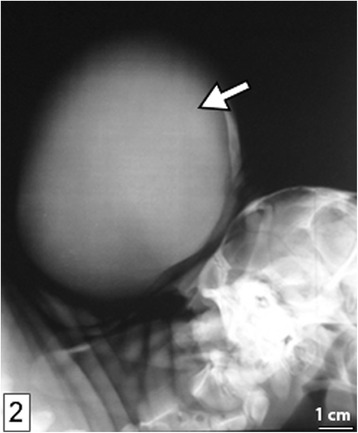
A portion of lateral head and neck radiograph showing the cystic sac (*arrow*) filled with homogenous content

The calf was always lying on lateral recumbency, with a roughly spherical fluctuant saclike protrusion on parieto-occipital region of the head, measuring 12x13cm and covered with hairy skin (Fig. 1). The calf exhibited resistance and groaned on deep palpation and neck flexion. Rectal temperature of 38.4 °C, respiration of 22 breaths per minute and pulse of 130 beats per minute were recorded. On deep palpation, the rim of the cranial bone defect could be felt around the base of the swelling. On palpation of the rostro-dorsal wall of the swelling, a hard, roughly wedge-shaped structure could be felt. Lateral head and neck radiographs revealed homogeneity of the swelling’s content suggestive of a fluid-filled cyst with no neural/brain tissue involvement (Fig. 2).

**Fig. 3:**
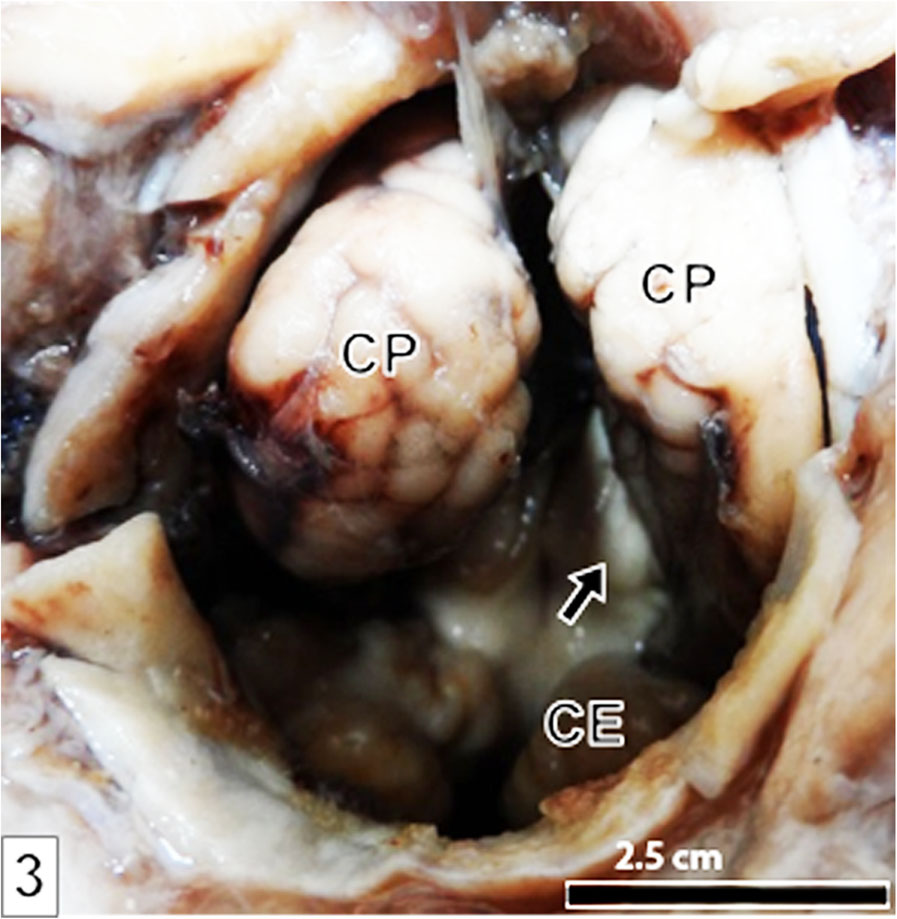
A photograph showing the caudal poles of cerebral hemispheres (CP) and portions of cerebellar hemispheres (CE) as visualized through the cranial bone defect after complete opening into the cyst. Notice the wide gap between the respective hemispheres of cerebrum and cerebellum and the widened transverse fissure exposing part of corpus quadrigermina (*arrow*)

**Fig. 4:**
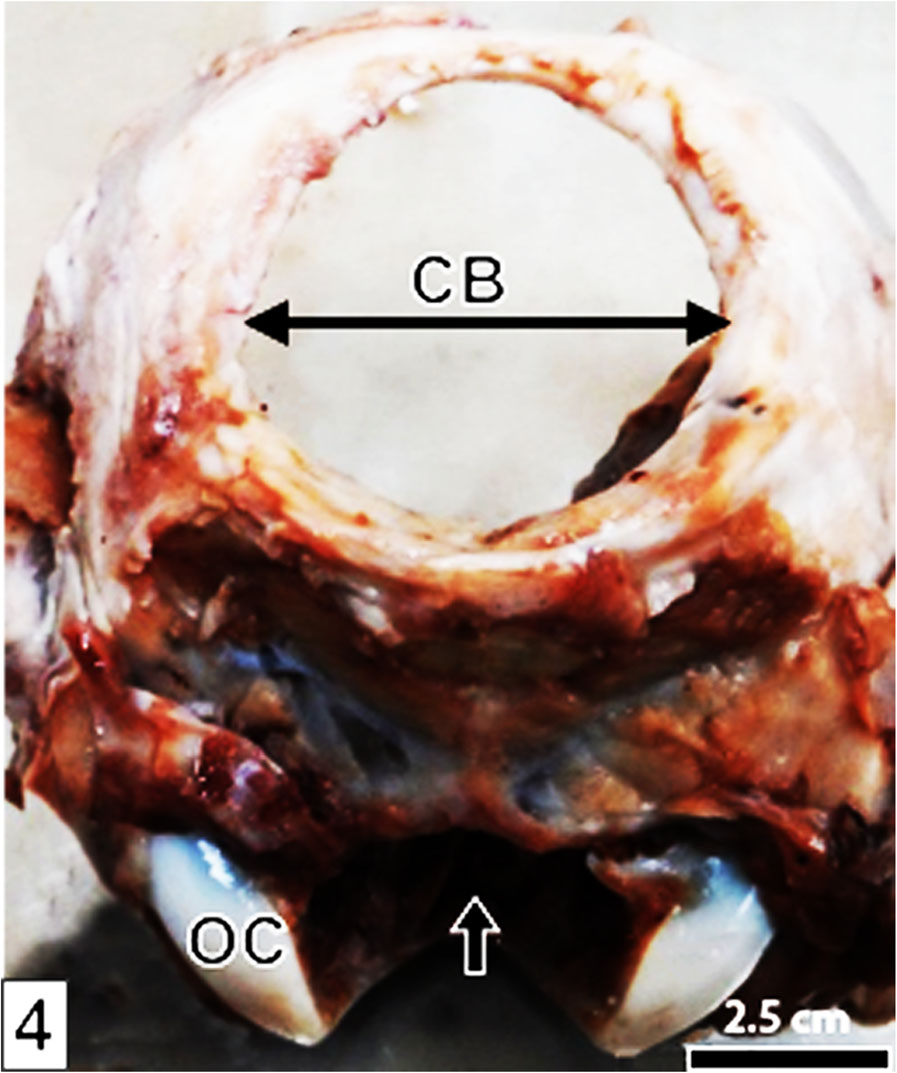
A photograph of the caudal part of the cranium after removal of the protrusion, flesh and brain. The circular cranial bone defect (CB), occipital condyles (OC) and entrance into foramen magnum (*arrow*) are shown

Straw-colored fluid, with glucose level of 8.2 mg/dl, was aspirated from the cyst. The calf was then euthanized followed by complete opening of the cyst where a total volume of 600 mL of liquid was obtained. The cyst was connected to the cranial cavity via an almost perfectly circular defect in parieto-occipital region of skull vault with a diameter of 5 cm and through which the caudal pole of the cerebral hemispheres and rostral part of cerebellum could be seen (Figs. 3 and 4). The cerebral hemispheres appeared wide apart while the cerebellum was displaced caudally so that transverse fissure was remarkably enlarged exposing part of corpus quadrigermina with the cerebellar hemispheres appearing abnormally wide apart.

**Fig. 5:**
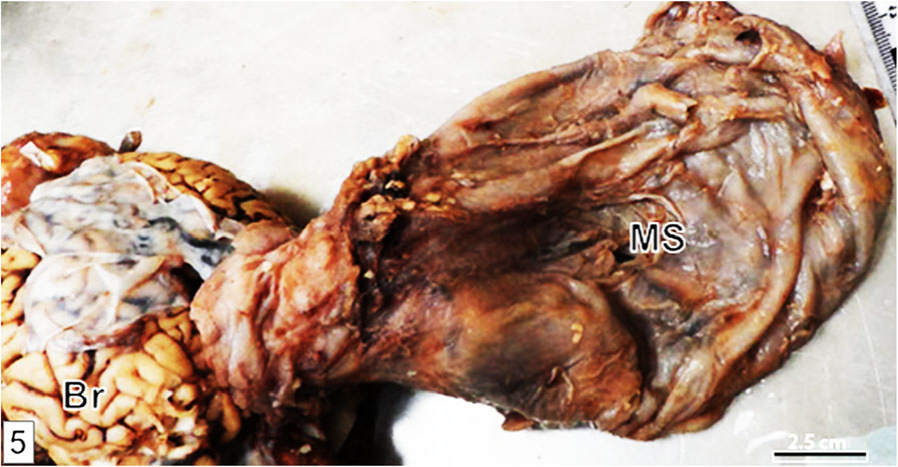
A photo of a drained meningeal sac (MS) after complete detachment from the skin and cranium. Notice its continuity with the meninges. Part of cerebrum (Br) is also shown

The liquid in the cyst was contained within a meningeal sac, which was relatively thick walled and adherent to the inner surface of the overlying hairy skin and to both the margin of the bone defect and inner surface of cranial bones. The sac was continuous with the brain meninges (Fig. 5) and its inner surface had features akin organ impressions, characterized by depressions separated by pillars of elevated tissue. Embedded to the wall of the meningeal sac was a narrow longitudinally oriented tongue-shaped bony structure. This structure measured 7.3 cm in length and was wider at the middle, measuring 1.8 cm, and then tapered gradually towards both extremities.

## Discussion

A number of congenital malformations have been reported in cattle and other domestic animal species with their development associated with either or a combination of hereditary and environmental factors [9, 12, 13, 15–17]. In meningocele or encephalomeningocele, the prime morphogenetic event that leads to the development of bone defects through which the meninges protrude, alone or together with neural tissue, has been traced to the focal failure of neural tube closure during fetal development [12, 14, 15]. This neural tube defect then precipitates failure in the development of skeletal encasement around the affected area of the neural tube [12]. These defects have mostly been reported to affect the frontal and occipital regions [17].

Craniomeningocele can be hereditary as has been reported in a number of domestic animals including cattle, sheep and pigs [11–13]. Environmental factors linked to the development of this condition are numerous, including chemical agents, such as griseofulvin when administered during pregnancy [11–13, 18]. Other environmental factors linked to the development of this congenital anomaly include malnutrition, infectious agents particularly viruses such as bovine viral diarrhea and poisonous plants among other involved causes [9, 11, 16, 19]. In addition, the application of excessive pressure during rectal examination of pregnant dam has also been suggested to predispose to this condition [11]. In the present case, the predisposing factor(s) could not be determined. A protrusion in the head region such as that in the present case, with detection of bone defect on deep palpation, could only be suggestive of either cranium bifidum with craniomeningocele or craninioencephalocoele. Radiographic examination revealed that the cyst’s content was homogenous, strongly suggesting that brain was not involved. Anatomopathologic examinations showed the presence of a circular bone defect in squamous part of occipital and that the protrusion did not involve the brain tissue, thereby confirming that it was a case of cranium bifidum with craniomeningocele and not cranioencephalomeningocele. The cystic sac, composed of an outer layer of hairy skin and an inner layer of dense connective tissue conforms to the description given in Parrah et al., [15].

Mammalian skull vault is mainly constructed by parietal and frontal bones with a contribution from squamous part of occipital bone on the caudal portion. The squamous part of occipital bone forms from fusion of supraoccipital with interparietal bones [1, 3, 6–8]. Interparietal bone in turn forms from fusion of elements derived from different ossification centers constituted by tissues of different embryonic origin [5–8]. These elements can in general be grouped into neural crest-derived median and mesoderm-derived lateral elements which are thought to represent mammalian homologs of post-parietal and tabular bones of non-mammalian tetrapods respectively [8]. The bonny structure embedded to the wall of the sac did not conform to the shape of defect and its source and significance could not accurately be established in this study. It can be hypothesized that this bony structure represent the median element of interparietal bone which, probably due to the defect and outward displacement by the cyst, failed to fuse with supraoccipital bone. This could therefore imply that neural crest cells, from which this component of squamous part of occipital bone is derived, migrated to establish ossification center for the bone notwithstanding the coinciding focal neural tube defect. The length of this structure was more than expected of the median component of interparietal in normal conditions. This may have resulted from overgrowth due to failure of its incorporation into squamous part of occipital bone and lack of contact with other developing bones due to its displacement. Presence of such a structure hasn’t been described in previous reports of cranial meningocele.

This indisposition can only be effectively treated by means of reparative surgery [10, 14]. Besides removing the protrusion and correcting the defect, surgical intervention essentially aims at relieving intra-cranial pressure which can have negative impact on brain development [10]. Therefore, for better chances of full recovery, surgical interventions should be effected early before the brain is substantially affected [10]. Such successful interventions have been reported in calves and a kid [10, 14, 16]. In the present case, the farmer could not access veterinary attention in his locality and it was rather late by the time a veterinarian from the faculty was called. It was the opinion of concerned veterinarians in the Faculty that the brain tissue had, most likely, been substantially affected and that chances of full recovery were slim. Furthermore, the farmer was not in a position to bear the cost of surgical intervention and post-operative management. For the interest of animal welfare, the concerned veterinary surgeons chose to euthanize the calf based on opinions that pressure due to accumulated fluid may have already impacted the brain and this was, most likely, manifested in the inability of the calf to stand on its own. In addition, the straw-color of the fluid indicated the calf was bleeding into the cyst.

In rural parts of Kenya and the rest of developing world, access to veterinary services is limited due to poorly developed private veterinary practice [20]. Besides unavailability of veterinarians in such regions, access to veterinary services is also limited by inability of farmers to pay for the clinical veterinary interventions [21]. For the case of livestock farmers, income from livestock products is generally low, due to low animal productivity and poor market prices for animal products [22, 23], and therefore often inadequate to pay for ‘costly’ veterinary services/interventions in the nature of the current case [24]. This in turn discourages establishment of private veterinary practices in their locality. Farmers, therefore, largely rely on state veterinarians or para-veterinarians who may not be available on time due to the vastness of their coverage area and poor facilitation. For this reason, cases that require urgent attention are often lost before arrival of a veterinarian or are adversely affected beyond recovery by the time the veterinarian arrives. In such a scenario, animal welfare is taken into consideration and euthanasia is often the only option.

## Conclusion

Anatomopathological findings confirmed that this was a case of cranial bifidum with meningocele. The defect affected parieto-occipital part of the skull vault and the bony structure embedded to the wall of the cyst was thought to, most likely, represent the median portion of interparietal bone, which, due to the defect, failed to be incorporated into squamous part of occipital bone. Presence of such a bony structure associated to the wall of the cyst is a rare finding which has not been reported in previous cases of cranial meningocele in animals.

## Abbreviations

°C: Degrees centigrade
cm: Centimeters
CSF: Cerebrospinal fluid
Mg/dl: Milligram per deciliter
mL: Milliliters
NTD: Neural tube defects
